# *Legionella pneumophila* p45 element influences host cell entry and sensitivity to sodium

**DOI:** 10.1371/journal.pone.0218941

**Published:** 2019-06-27

**Authors:** Lanette M. Christensen, Preeti Sule, Madison Strain, Jeffrey D. Cirillo

**Affiliations:** Department of Microbial Pathogenesis and Immunology, College of Medicine, Texas A&M University Health Science Center, Bryan, Texas, United States of America; Purdue University, UNITED STATES

## Abstract

*Legionella pneumophila* are environmental bacteria found ubiquitously in both natural and man-made water reservoirs, sometimes as constituents of biofilm communities, but mostly intracellularly within protozoal hosts. In the event that *Legionella* become aerosolized in water droplets and inhaled by humans, they can cause a potentially fatal form of pneumonia called Legionnaires’ disease. Strains of *L*. *pneumophila* have highly plastic genomes that harbor numerous inter- and intra-genomic elements, enhancing their ability to live under diverse environmental conditions. One such mobile genomic element, p45 carries ~45 kbp of genes, including the Lvh (Legionella Vir homolog) type IVa secretion system. This element was evaluated for its contribution to *L*. *pneumophila* environmental resilience and virulence-related characteristics by comparing clinically isolated strain Philadelphia-1 that carries p45, Lp01 that lacks p45, and Lp01 with p45 reintroduced, Lp01^+p45^. We found that the p45 element impacts host cell entry and resistance to sodium, both virulence-related characteristics in *Legionella* species.

## Introduction

*Legionella pneumophila* are Gram negative facultative intracellular bacilli that ubiquitously inhabit water reservoirs [1–6]. These environmental bacteria often live as constituents of biofilm communities or intracellularly within protozoal hosts [1, 2]. Coevolution with single celled eukaryotic hosts presumably equipped these bacteria with the ability to also utilize human alveolar cells as hosts [7–10]. Humans can become incidental hosts to *L*. *pneumophila* when the bacteria are aspirated as contaminated aerosol droplets [11, 12]. Infected individuals can then develop a potentially fatal form of acute pneumonia called Legionnaires’ disease, or an acute self-limiting flu-like illness called Pontiac fever [13]. Not all *L*. *pneumophila* strains exhibit the same ability to cause severe human disease [14, 15]. It has been suggested that differences in the ability of different strains to cause clinical disease are not due to environmental prevalence, but that virulence of different *Legionella* strains vary [16–18]. Our prior studies found that there are specific genetic loci, including mobile genetic elements, that correlate with clinically relevant *Legionella* strains and some of these loci are differentially regulated by temperature [16, 19].

*L*. *pneumophila* are capable of withstanding vastly diverse environmental conditions in the water environments that they are found within, even when they have drastic temperature fluctuations [20]. Efficient survival in the environment is aided by intricate regulatory systems [21, 22] and plasticity of their genome [23, 24]. The *L*. *pneumophila* genome varies greatly among strains due to the prevalence of inter and intra-genomic mobile elements [25]. One such class of genomic elements, called mobile integrative elements (MIE), are capable of existing as extra-chromosomal (episomal) circular plasmid-like structures or integrated site-specifically in the chromosome [26, 27]. Distinguishing features of mobile integrative elements include different G+C content than the chromosomal DNA, often flanked by tRNA, and frequently encode phage-related genes and conjugation machinery [23, 28]. These elements commonly have the capacity to transfer from one bacterium to another via conjugation adding to the dynamic plasticity of the *Legionella* genome [23–25, 27]. Elements that have both the ability to be conjugated and integrate in the genome are referred to as integrative conjugative elements (ICE) [23–25, 27].

An example of a MIE in the *L*. *pneumophila* serogroup 1 strain Philadelphia (Phil-1) is the p45 element, which we recently found can be conjugated, making it an ICE. p45 includes all of the features common to MIEs and ICEs and encodes the *Legionella* Vir homolog (Lvh) type IVa secretion system (T4aSS) [16, 19] along with the *lvr* gene cluster that includes a homolog of the CsrA global regulator, LvrC [29]. Recently, p45 was shown to impact *L*. *pneumophila* virulence in the guinea pig model. Although it is possible that p45 has a role in environmental reservoirs as well as during disease, the function(s) of p45 in different environments are not well understood.

In the current study, the *L*. *pneumophila* ICE p45 was evaluated for its role in different environmental conditions and during interactions with host cells by comparing the wild type strain Phil-1 that naturally carries p45 [30] with strain Lp01 that does not carry p45 [16, 31] and a strain of Lp01 where the p45 ICE has been returned to the strain (Lp01^+p45^). We found that although p45 does not appear to impact growth in vitro, it has an impact upon susceptibility to the environmental stress from NaCl. Acidic pH had a greater effect on viability of Lp01 than Phil-1, but this effect was not corrected by p45, suggesting that other differences between Phil-1 and Lp01 are responsible for greater susceptibility to acidic environments. Similarly, the effect of reactive oxygen species and high temperature on bacterial viability were not affected by p45 and pigmentation levels were similar. However, p45 does appear to play a role during entry into host cells, both the environmental amoeba *Acanthamoeba castellanii* and the murine macrophage cell line J774A.1. These observations suggest that the p45 ICE is important for survival of *Legionella* in high salt environments and efficient infection of host cells that serve as the replicative compartment both during disease and in the water environments.

## Results

### Involvement of p45 in environmental stress resistance

**Fig 1:**
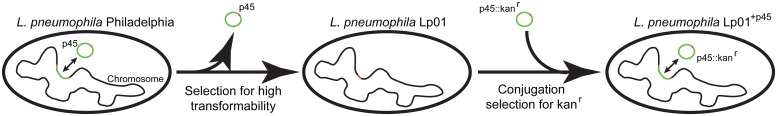
Characteristics of *L*. *pneumophila* strains. The original *L*. *pneumophila* serogroup 1 strain Philadelphia carries the p45 element (green) that is both integrated in the chromosome and episomal, with the episomal copy number at approximately three copies per copy of the bacterial genome. The attachment sites for site-specific recombination in the chromosome and on p45 are shown in red. Upon selection for a strain that displays a high efficiency of transformation, the p45 element was lost, creating strain Lp01. The p45 element was returned to Lp01 by conjugation from a strain of Philadelphia that had been modified to express kanamycin resistance (kan^r^) from p45. The resulting strain of Lp01 was confirmed to carry p45 and maintain all chromosomal markers associated with strain Lp01, including point mutations in *luxN* and *rpsL* (confers streptomycin resistance) and a 9 bp deletion within *ndh*, by PCR and sequencing.

**Fig 2:**
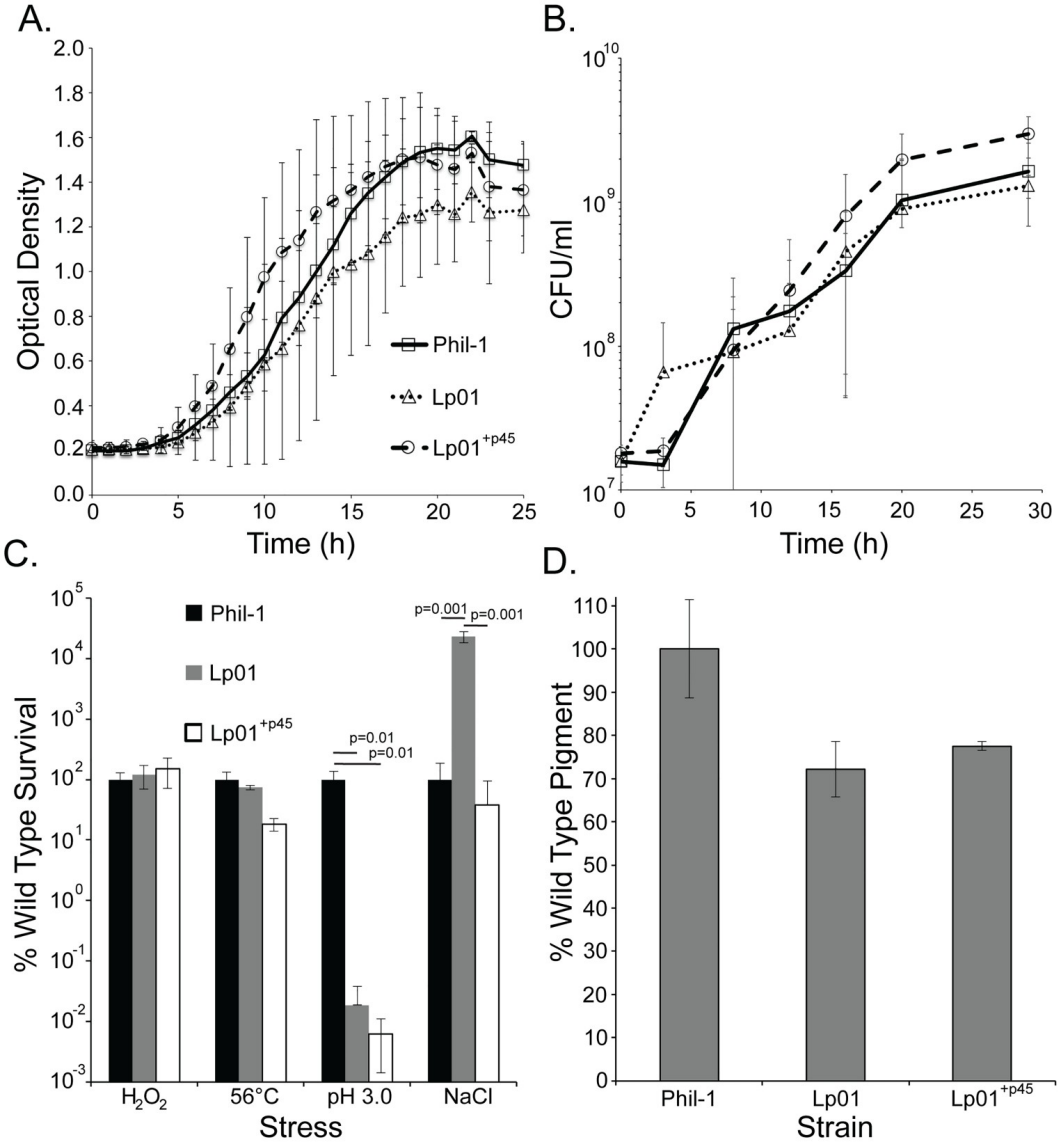
Effects of environmental stressors. Growth of *L*. *pneumophila* serogroup 1 strain Philadelphia (Phil-1), Lp01, and Lp01 containing p45 (Lp01^+p45^) in BYE liquid media as measured by (A) optical density at 600 nm (OD_600_) and (B) colony forming units (CFU). (C) Percent survival after 30 min treatment with H_2_O_2_, 56°C, acidic pH (pH 3.0) and 1% NaCl in BYE liquid medium with shaking (210 rpm). Percent wild type survival was calculated as the number of bacteria from each strain remaining after treatment divided by the number of bacteria in medium without treatment relative to wild type (Phil-1), with wild type set to 100%. Data are representative of results in three independent experiments executed in triplicate. (D) Pigment production was measured in the supernatant of *L*. *pneumophila* Phil-1, Lp01, and Lp01^+p45^ grown 32 h in BYE liquid media. Percent wild type pigment was calculated as the pigment produced by the strain relative to wild type (Phil-1), with wild type set to 100%. Supernatants were examined for pigment production every 4 h until 32 h. Pigment production was not significant until the 32 h time point that is shown. Data for pigment production represent results of two independent experiments in triplicate. Data points are means and error bars represent standard deviations.

A variant of the wild type strain Phil-1 that could be easily transformed and was streptomycin resistant (Fig 1) was previously selected to facilitate laboratory studies on this strain and was designated Lp01 [32]. We moved the p45 ICE back into Lp01, creating Lp01^+p45^, by conjugation from a strain of Phil-1 that carried p45 marked with kanamycin resistance. Growth of Phil-1, Lp01 and Lp01^+p45^ were similar in laboratory media, in both the liquid medium BYE and solid BCYE agar as lawns, displaying no significant differences in growth for any of our experimental conditions (Fig 2A and 2B). However, Lp01^+p45^ was compared with Lp01 and Phil-1 for the ability to survive under stressful environmental conditions. Conditions tested included heat (56°C), H_2_O_2_, acidic pH, and presence of sodium. No significant morphological differences were observed in the presence of any of these stressors by phase contrast microscopy. Sensitivity to sodium has been used for decades in the *Legionella* field as an *in vitro* proxy for virulence, where strains more sensitive to sodium tend to be more virulent [16, 33, 34]. The Phil-1 strain and Lp01^+p45^ display similar levels of sensitivity to sodium, while Lp01 was significantly more resistant (Fig 2C). Resistance to heat and the presence of H_2_O_2_ does not differ between the three strains, while Phil-1 recovered better than the Lp01 and Lp01^+p45^ strains after acidic pH stress (Fig 2C).

### Pigmentation production

The three *L*. *pneumophila* strains were evaluated for their ability to produce pigmentation, which provides an advantage for bacteria living in the environment and is expressed in stationary phase [35]. Similar levels of pigmentation were detected in Lp01 and Lp01^+p45^ cultures. Pigment production was not significant until 32 h where Phil-1 produced pigment in the supernatant that appeared to be at slightly higher levels (Fig 2D), but this apparent difference did not reach statistical significance (P = 0.09). Considering that there are no significant differences in growth of these strains, the increased pigment production in Phil-1 culture would be due to differences between this strain and Lp01, but not due to the p45 ICE, similar to the effects of an acidic environment.

### Involvement of p45 in host cell entry

**Fig 3:**
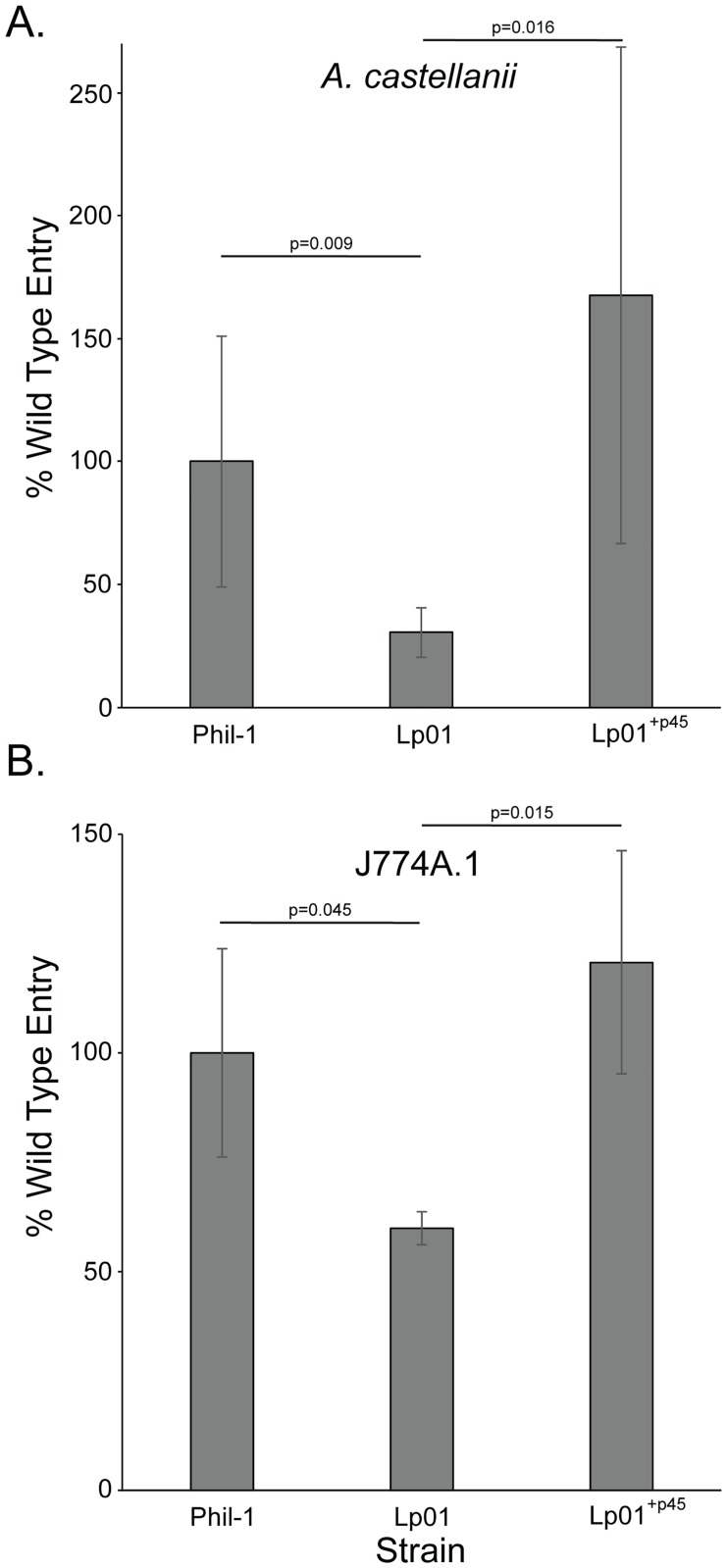
*Legionella pneumophila* entry into host cells. The environment protozoal host (A) *A*. *castellanii* amoeba and mammalian (B) J774A.1 murine macrophages were infected with *L*. *pneumophila* Phil-1, Lp01, or Lp01^+p45^ for 30 minutes at an MOI ~50 and ~10, respectively. Percent wild type entry was calculated as the number of bacteria that became gentamicin resistant divided by the inoculum relative to wild type (Phil-1), with wild type set to 100%. Data represent results from three independent experiments executed in triplicate. Data shown are means and error bars represent standard deviations.

Previous studies in our laboratory found that p45 carries temperature regulated genes that impact interactions with host cells [19], making it likely that p45 plays a role in host cell infection. Phil-1, Lp01, and Lp01^+p45^ were examined for their ability to enter the environmental amoeba *A*. *castellanii* and mammalian J774A.1 cells, which is a murine macrophage cell line. Infection of *A*. *castellanii* revealed that strain Lp01 does not enter these cells as well as Phil-1 (Fig 3A). Introduction of p45 in Lp01^+p45^, rescued the ability of Lp01 to enter amoebae. When used to infect the murine macrophage cell line J774A.1, similar results were obtained to those in amoebae (Fig 3B). The strain Lp01 did not enter host cells as well as either the wild type strain Phil-1 or Lp01^+p45^. Although it is possible that the same temperature regulated loci identified in our prior studies play a role in these host cell interactions, all assays were carried out at 37°C and the bacteria were grown at 37°C indicating that p45 plays a role in host cell entry even at 37°C. Together these data suggest involvement of p45 ICE in entry into host cells. It is likely that p45 would impact the prevalence of *Legionella* in the environment and its ability to cause disease in humans.

## Discussion

Phenotypic differences between *L*. *pneumophila* Phil-1 and Lp01 that were rescued by the presence of p45 in strain Lp01^+p45^ include entry into J774A.1 monocytic cells and amoebae and sensitivity to sodium. Since sodium sensitivity represents a good proxy for virulence [33, 34], more virulent strains of *Legionella* are likely to exhibit greater sensitivity to sodium. Although a mechanistic explanation for this association is not fully understood, at least in some cases the defect has been localized to *dotA*, involved in type IV secretion, that is known to also impact sensitivity to sodium [34, 36, 37]. Prior studies have also demonstrated a relationship between sodium sensitivity and the DotU and IcmF subunits of the Dot/Icm type IVb secretion system [38]. Interestingly, there are a number of Dot/Icm components that share similarities with those of the Lvh type IVa secretion system [39], presenting a likely explanation for the increased resistance to sodium displayed by Lp01 in comparison to strains with p45. Regardless of mechanistic explanation, increased sodium sensitivity displayed by Phil-1 and Lp01^+p45^ suggests the presence of p45 is associated with *L*. *pneumophila* virulence, as inferred by our prior observation that the *lvh* type IVa secretion correlates with virulence and improved intracellular growth in human monocyte-derived macrophages and amoebae [16].

Regulation of sodium resistance is impacted by the global regulator, CsrA [21]. *L*. *pneumophila* possess four homologs of CsrA and one of these is encoded on p45, *lvrC* [29, 39]. The *lvrC* (*lpg1257*) gene maintains 39% identity with *csrA* (*lpg1593*) [29], is located within the lvh/lvr region, and is believed to be involved in regulation of Lvh [29]. Phenotypes known to be regulated by *Legionella csrA* (*lpg1593*) include pigmentation, motility, and resistance to stressful conditions such as acidic pH, heat, osmotic pressure, sodium, and H_2_O_2_ [21]. When tested, phenotypes such as heat, H_2_O_2_, and acid resistance did not correlate with *L*. *pneumophila* strains that carry p45. Susceptibility to sodium stress was the only *csrA*-associated phenotype tested that correlated with the presence of the p45 ICE, suggesting that *lvrC* does not greatly impact the same phenotypic characteristics of the *Legionella csrA* gene and may play other roles during disease or in the environment. All of our stress studies used *Legionella* grown on BCYE agar and our prior studies show *Legionella* phenotypic characteristics can vary greatly depending upon growth conditions [10, 16, 40], making examination of additional conditions important to better understand how the genes involved are expressed in the environment. Examining differences in concentrations or level of each stress could also provide insight into subtle differences between these strains that were not apparent under the conditions used in the current study. The phenotypic differences between Phil-1 and Lp01 that were not recovered by reintroduction of p45, acid resistance and pigment production, may be associated with one or more of the other known genetic differences between Lp01 and Phil-1 [31, 41]. In particular, the point mutation in the putative sensor histidine kinase *luxN* or the 9 bp deletion in the putative NADH dehydrogenase *ndh* could impact these phenotypic characteristics. Although either of these genes could play a role in the acid resistance and pigment production phenotypes, there have not been any studies examining the biological roles of these genes in *Legionella*, making speculation regarding their roles premature.

The ability of *L*. *pneumophila* to enter both mammalian and protozoan host cells is impacted by the p45 ICE. Entry into the environmental host *A*. *castellanii* was influenced by the presence of p45 in similar manner to that observed in the murine macrophage cell line J774A1. These observations are consistent with our previous studies indicating that entry into both environmental and mammalian host cells can occur by similar mechanisms [9, 10, 16, 19, 42–44]. This phenotype might suggest that this host cell infection advantage was selected for in the environment and that the ability to infect mammalian cells conferred by p45 ICE is a consequence of evolution to increase the efficiency of infecting protozoa. It should be taken into account that *A*. *castellanii* are not the only environmental hosts for *Legionella*. In fact, there are numerous protozoan species that can serve as hosts, including *Vermamoeba* (*Hartmanella*), *Naegleria* and *Tetrahymena* as well as most likely many others that have not yet been well characterized [12]. *Legionella* strains interact and parasitize various protozoal hosts with different efficiencies, but the extent of this variance is not known. Therefore, the effects on host cell infection observed in our studies may not necessarily be consistent among all protozoa capable of serving as replicative hosts for *Legionella* and these alternative hosts may have unknown and/or inconsistent impacts on interactions with mammalian hosts. Furthermore, we could not extensively test all environmental conditions and further work is needed to examine those that are likely to play a role in regulation of p45, including temperature [19]. The presence of additional regulatory elements that are known to impact virulence-related characteristics, including noncoding RNA [45], makes investigation of the global regulatory consequences of the p45 ICE important for understanding pathogenesis of *Legionella*.

Collectively, our observations suggest a role for the p45 ICE in *Legionella pneumophila* host cell infection and susceptibility to environmental salt conditions. These phenotypic characteristics suggest that the p45 ICE contributes to *L*. *pneumophila* survival in the environment and could impact the ability of these bacteria to cause disease. We are particularly interested in whether the distribution of the p45 correlates with strains of *Legionella* that are more likely to cause infections that lead to clinical symptoms and are responsible for larger epidemics. Examination of the distribution of p45 ICE in environmental and epidemic-associated isolates is warranted to better elucidate its role in the epidemiology of Legionnaires’ disease.

## Methods

### Bacterial strains and growth conditions

*Legionella pneumophila* Philadelphia-1 (Phil-1) and its derivative Lp01 were kindly provided by both Ralph Isberg and Michelle Swanson. Lp01 is a streptomycin-resistant variant and restriction deficient mutant of the clinical strain Phil-1 [32]. Strain Lp^+p45^ was constructed by conjugating a p45 modified to carry kanamycin resistance (*aph*) into strain Lp01. *Legionella* strains were grown on buffered charcoal yeast extract (BCYE) agar plates [46] or shaken in buffered yeast extract (BYE) broth, and kept at 37°C and 5% CO_2_ for 3–5 days. *L*. *pneumophila* used for experiments were grown from 4°C stock lawns, which were grown from -80°C glycerol stocks. Stock lawns were kept and used at 4°C for no longer than 5 days. No strains were passed more than three times in the laboratory, to ensure full virulence was maintained.

### Protozoal strains and growth conditions

*Acanthamoeba castellanii* (ATCC 30234), an environmental host for *L*. *pneumophila* [47], was maintained in M712 media at 22°C in 75-cm^2^ tissue culture flasks in the dark as previously described [9, 10]. Prior to use, *A*. *castellanii* were seeded in 24-well plates at 5 x 10^5^ cells/well for 12 hours, the media was then washed and replaced with M712 medium without proteose peptone and yeast extract and incubated at 37°C and 5% CO_2_ for 1 hour. Amoebae viability and enumeration were determined using Trypan Blue staining and a hemocytometer. No significant differences in amoebae viability were observed in our studies, with viability ranging from 98–99% in all experiments.

### Cell culture, strains and growth conditions

The mouse cell line J774A.1 was maintained in 75-cm^2^ tissue culture flasks with RPMI 1640 plus 2mM L-glutamine and 5% heat inactivated fetal bovine serum at 37°C and in 5% CO_2_, as described previously [48]. Tissue culture cells were seeded in 24-well plates at 2.5 x 10^5^ cells/well 18 h prior to use. Trypan Blue staining and a hemocytometer were used to determine cell numbers and viability. No significant differences in macrophage viability were observed in our studies, with viability ranging from 95–98% in all experiments.

### Stress assays

Cultures of *L*. *pneumophila* Phil-1, Lp01, and Lp01^+p45^ grown as lawns on BCYE were suspended in BYE alone or BYE containing a stress treatment, at an OD_600_ of ~0.1. Stress treatments included 1% (w/v) NaCl, pH 3 citrate, 56°C, and 3 mM H_2_O_2_ as previously described [49]. In all cases, the concentrations of bacteria used for assays were the same initially for each strain. Cultures were incubated with 210 rpm shaking at 37°C for 30 minutes (with the exception of the heat stress samples, which were kept at 56°C in BYE) before diluting and plating the samples on BCYE which were then incubated at 37°C and 5% CO_2_ for 3–5 days. Colony forming units (CFU) that arose from cultures grown in BYE alone at 37°C were compared with those from the stress treated samples and the results were calculated as percent resistance relative to wild type (Phil-1), with wild type resistance set to 100%. Phase contrast microscopy was conducted on *Legionella* by suspending samples in BYE medium and placing drops from suspensions directly on slides with coverslips prior to visualization.

### Pigmentation assays

Pigment production was monitored by optical density (550 nm) of the culture supernatant as described previously [21, 35]. *L*. *pneumophila* Phil-1, Lp01, or Lp01^+p45^ grown on BCYE was inoculated to an optical density of 0.2 (600 nm) and shaken (210 rpm) at 37°C for 32 hours. Every four hours the optical density (550 nm) of the supernatant was measured.

### Growth rate in liquid media

*L*. *pneumophila* Phil-1, Lp01, and Lp01^+p45^ were inoculated into BYE broth and shaken (210 rpm) at 37°C for 32 hours. Optical density (OD_600_) measurements at 600 nm were determined every half hour and the number of viable bacteria was quantified by CFU on BCYE agar plates every 3 hours. OD_600_ readings were taken at 600 nm in a spectrophotometer using disposable sterile cuvettes and dilutions of cultures made until readings fell between 0.1 and 1.0 to allow accurate density measurement and calculations made to determine the OD of the original culture.

### Entry assays into host cells

Entry assays were conducted essentially as described previously [16, 30]. Briefly, *L*. *pneumophila* cultures grown on BCYE plates were suspended and diluted in the same medium as used to grow the host cells to be infected. Bacteria were added to 24-well plates containing 10^6^ J774A.1 cells [30] or *A*. *castellanii* strain Neff [10] at a multiplicity of infection of 10 or 50, respectively. Following 30 minutes of co-incubation with the bacteria, wells were washed twice and then incubated for 2 hours with the appropriate medium containing 100 μg/ml of gentamicin, then washed twice more. Host cells were lysed in sterile ddH_2_O and the number of intracellular bacteria was determined by CFU on BCYE plates. Percent entry was then determined by dividing the CFU of intracellular bacteria by the CFU used for infection, then multiplied by 100.

### Statistical analysis

The significance of the results was determined using Student’s T-test or analysis of variance, as appropriate. All data obtained in these studies were normally distributed, as expected. All studies were conducted in triplicate and repeated at least twice with similar results obtained. Data from representative experiments are provided. P values less than 0.05 were considered significant. Microsoft Excel V15.26 and GraphPad Prism V5 software were utilized for statistical analysis.
